# Cancer treatments touch a wide range of values that count for patients and other stakeholders: What are the implications for decision‐making?

**DOI:** 10.1002/cam4.5336

**Published:** 2022-11-14

**Authors:** Cilla E. J. Vrinzen, Haiko J. Bloemendal, Esra Stuart, Amr Makady, Michel van Agthoven, Mariska Koster, Matthias A. W. Merkx, Rosella P. M. G. Hermens, Patrick P. T. Jeurissen

**Affiliations:** ^1^ Scientific Institute for Quality of Healthcare (IQ Healthcare), Radboud Institute of Health Sciences (RIHS) Radboudumc Nijmegen The Netherlands; ^2^ Netherlands Comprehensive Cancer Center (IKNL) Utrecht The Netherlands; ^3^ Department of Oncology, Radboud Institute of Health Sciences (RIHS) Radboudumc Nijmegen The Netherlands; ^4^ Janssen‐Cilag B.V. Pharmaceutical Companies of Johnson & Johnson Breda The Netherlands

**Keywords:** clinical decision‐making, decision‐making, neoplasms, patient preference, quality of life, quality improvement, shared

## Abstract

**Background:**

Cancer rates and expenditures are increasing, resulting in debates on the exact value of this care. Perspectives on what exactly constitutes worthwhile values differ. This study aims to explore all values–elements regarding new oncological treatments for patients with cancer and all stakeholders involved and to assess their implications in different decision‐making procedures.

**Method:**

Thirty‐one individual in‐depth interviews were conducted with different stakeholders to identify values within oncology. A focus group with seven experts was performed to explore its possible implications in decision‐making procedures.

**Results:**

The overarching themes of values identified were impact on daily life and future, costs for patients and loved ones, quality of life, impact on loved ones, societal impact and quality of treatments. The expert panel revealed that the extended exploration of values that matter to patients is deemed useful in patient‐level decision‐making, information provision, patient empowerment and support during and after treatment. For national reimbursement decisions, implications for the broad range of values seems less clear.

**Conclusion:**

Clinical values are not the only ones that matter to oncological patients and the stakeholders in the field. We found a much broader range of values. Proper recognition of values that count might add to patient‐level decision‐making, but implications for reimbursement decisions are less clear. The results could be useful to guide clinicians and policymakers when it comes to decision‐making in oncology. Making more explicit which values counts for whom guarantees a more systematic approach to decision‐making on all levels.

## 1 INTRODUCTION

The worldwide cancer rate is expected to increase from 19 million in 2020 to 30 million in 2040.^1^ Costs of cancer care are high and continue to rise. In Europe, costs have almost doubled from €52 billion in 1995 to €103 billion in 2018.^2^

Over the years, patient‐centred care is gaining increasing interest in which the needs and desires of individual patients drive the force behind healthcare decisions and quality measurements.^3^, ^4^, ^5^, ^6^ For payors and policymakers, it is challenging to provide optimal healthcare needs for individual patients while managing budgets. Decision‐making on reimbursement and resource allocation is often aided by using Health Technology Assessment (HTA).^7^, ^8^ However, HTA frameworks do not always reflect all values that count for patients and other stakeholders, therefore, various different value frameworks have been published in recent years,^9^, ^10^, ^11^, ^12^ for instance, by the American Society of Clinical Oncology (ASCO)^13^ and European Society for Medical Oncology (ESMO).^9^ Previous studies compared such oncological value frameworks and revealed some inconsistencies which mainly derived from the differences in perspective.^14^, ^15^, ^16^, ^17^ Additionally, it is argued that they do not take the unique aspects of the evolving therapeutic landscape with targeted therapy, immunotherapy and more precision medicine into consideration, which has led to increasing uncertainty about the true value of new treatments.^18^, ^19^

The International Society for Pharmaceutical Outcomes Research (ISPOR) Value Assessment Frameworks Special Task Force emphasized gaps in value assessment in general as elements from a societal perspective are missing, for example, equity.^20^, ^21^ It is also argued that the commonly used quality of life (QoL) outcome measures do not always seem adequate for mapping all aspects in the social domain^22^; they contain a subset of relevant outcomes.^21^, ^22^, ^23^, ^24^, ^25^ Over the years, different disease‐specific QoL measurement tools have been developed.^26^

What exactly constitutes value to whom in the context of cancer treatments, limited financial budgets and patient‐centred care is still not clear and varies between different stakeholders. The aim of this study is therefore to explore values–elements regarding new oncological treatments and to assess their implications in decision‐making procedures to inform the oncological field and generate the maximum value with fixed national budgets.

## 2 METHOD

### 2.1 Study design

We conducted semi‐structured interviews to explore values regarding new oncological treatments from different stakeholder perspectives. Values are defined as elements that warrant consideration in treatment assessment and decision‐making procedures. In addition, a focus group with an expert panel was performed to discuss the usefulness and potential implications of the values that were found in the interviews in different decision‐making procedures. The study was reported based on recommendations in the consolidated criteria for reporting qualitative studies (COREQ) checklist.^27^

### 2.2 Study setting

This study was performed in the Netherlands. See Box 1 for a description of the Dutch healthcare system and see Kroneman et al. for a more extended description.^28^

**BOX 1:** Description of the Dutch Healthcare system

The Dutch government pursues three main goals for the healthcare system: quality (effective, safe and patient‐centred), accessibility and affordability. The *Netherlands Ministry of Health* (*MOH*) supervises healthcare and is responsible for access, quality and cost in the health system, has overall responsibility for setting priorities and can introduce legislation. The *National Healthcare Institute* (*Zorginstituut Nederland*, *ZIN*) assesses new technologies on (cost‐)effectiveness and advises the minister on the reimbursement and uptake into the insurance benefit package. Drugs below a certain cost threshold (e.g. budget impact of less than €10 million per year) that are proven to have an added therapeutic effect are immediately admitted into the benefits package. Above certain price thresholds, the ZIN assess new drugs on necessity, effectiveness, cost‐effectiveness and feasibility. The health minister makes the final decisions for these drugs above certain cost thresholds and performs price negotiations with pharmaceutical companies. In recent years, the movement towards patient‐centred care is stimulated by the government and different professionals. All residents in the Netherlands are obliged to have a basic health insurance package and pay a minimum of 385 Euro on statutory deductible premiums annually. People with lower incomes often receive a care allowance to reduce financial difficulties. In addition, patients can choose complementary insurance, for example extra cover for physical therapy.

### 2.3 Study population

To identify values within oncology, 12 stakeholder groups were defined for interviews: patients, partners of patients, oncologists, oncological nurses, occupational doctors, general practitioners, insurance advisors, the National Healthcare Institute (ZIN), Netherlands Comprehensive Cancer Center, the Ministry of Health (MOH), Health insurance companies and dedicated oncological care networks. From each group, participants for the interviews were purposively sampled to ensure a minimum of two interviews. We approached 41 potential participants by email through our investigator network, stakeholder websites and snowball sampling.

To discuss the usefulness and potential implications of the values in decision‐making procedures, an expert panel was purposively sampled with experts on quality of life, ethics, HTA and spokesmen of patients, ZIN, health insurance companies and healthcare professionals. Eleven experts were approached.

Stakeholder selection was based on screening of Dutch policy documents and literature and consultation with experts. The interviews and expert panel meetings were performed by video calls, and informed consent was obtained from all study participants.

### 2.4 Data collection

For the interviews, we drafted a topic guide (Appendix A) on overarching themes found in a literature search (Appendix B). These themes were societal impact, quality of life, impact on daily life, family burden, costs and quality of care. We applied an iterative approach in which, depending on the stakeholder and after reflections, slight adjustments or more emphasis on specific topics were made during the interviews. One trained researcher (CV) conducted the interviews.

The topic guide of the expert focus group (Appendix C) was constructed based on findings from the interviews and contained questions regarding the desire and current use of values in decision‐making procedures, within which context, it was desirable and with which methods values could be best measured. The expert panel was moderated by an experienced researcher (RH) and planned for 60 minutes.

### 2.5 Data processing and analysis

Interviews and the focus group with the expert panel were audio‐recorded and transcribed verbatim. The transcripts were thematically analysed. Codes were assigned to relevant text passages and codes referring to the same underlying concept were divided into subcategories and themes. Twelve transcripts, one from each stakeholder group, were coded by two trained researchers individually (CV and ES). After comparing transcripts, the researchers reached a consensus on the codes, subcategories and themes. The remaining transcripts were assessed by one researcher (CV) and discussed with the second researcher (ES) until a consensus was reached. All generated themes and subcategories are reported and listed indiscriminatory in the results. Atlas.ti (version 8) was used for data processing and analyses.

## 3 RESULTS

For the interviews, 41 stakeholders were approached of which 32 participated in 31 separate interviews (Table 1) with at least two interviews per stakeholder group. Of the non‐participators, one declined based on time constraints, six did not respond to email contact and two believed not to be the right person for this study. The interviews lasted between 20 and 64 minutes.

**TABLE 1:** Overview of stakeholders interviewed

Stakeholders	*N*
Patient (association)	
Ex‐patient	2
Patient association	1
Partner of patient	2
Oncologists	4
Oncological nurse	2
Occupational/company doctor	
Company doctor	1
Oncological occupational physician	1
General practitioners	3
Insurance advisor	
Intermediary insurance advisor (for life insurance)	2^a^
Re‐insurance^b^ advisor for life insurance companies	1
Employee Insurance Agency Netherlands (UWV)	1
National Healthcare Institute (Zorginstituut, ZIN)	2
Netherlands comprehensive cancer organization	2
Ministry of Health (MOH)	2
Health insurance companies	2
Oncological care networks	
Primary care network	1
Secondary care network	2
Chain management	1
Total	32

^a^ One interview with two participants.

^b^ Insurance companies can re‐insure themselves for high‐risk clients.

For the focus group, seven experts participated: an expert on health‐related quality of life, ethics, health technology assessment and a spokesman of a patient organization, the ZIN, a health insurance company and an oncologist. Of the non‐participators, one was not available on the date of the expert panel and three did not feel able to contribute.

### 3.1 Interviews stakeholders

The themes generated from the interviews were impact on daily life and future, costs for patients and loved ones, quality of life, impact on loved ones, societal impact and quality of treatments. The themes and subcategories generated from the interviews are presented in Table 2. Quotes from the interviews are presented in Table 3.

**TABLE 2:** Themes and subcategories from the interviews

Impact on daily life and future of patients
Daily functioning
Time investment
Impact on future plans and choices
Ability to:
work
do volentary work
perform hobbies
perform household chores
participate / contribute to society
be an informal caregiver
receive a mortgage
sport
care for children
be independent / self‐sufficient
Costs for patients and loved ones
Costs:
Reintegration
Disability insurance premiums
Additional care
Statutory deductible premium
New wardrobe
Medical devices
Prosthesis, swimwear, bra
Wigs
Home renovation
Childcare
Life insurance premiums
Travel expenses patients/relatives
Loss of income
Additional patient costs
Family costs
Quality of life—physical
Physical functioning:
Weakened immune system
Amputation
Physical capacity
Osteoporosis
Bowel functioning
Dry mucous membranes
Eating disabilities
Hearing damage
Weight changes
Joint pain
Hair loss
Cardiovascular diseases
Neuropathy
Edema
Infertility
Pain
Sexual functioning
Muscle strength
Loss of speech
Stoma
Change in taste
Fatigue
Early menopause
Skin irritation
Fever
Body integrity
Physical fitness
Scarring issues
Nausea
Quality of life—psychological
(Long‐term) Emotional functioning:
Acceptance
Fear
Fear of recurrence
Anger
Cognition and concentration
Confrontation
Loss of control
Depression
Dealing with dying
Loneliness
Frustration
Behavioral changes
Feeling good
Hope
Childlessness
Feeling like a burden
Misunderstanding by others
Worry about future health
Preserving positive attitude
PTSD
Sadness
Anxiousness
Change in attitude
Moving on
Trust in body
Self Confidence
Worry for loved ones
Need for companionship
Quality of life—social
Social functioning
Relationship with (grand)children
Relationship with partner
Relationship with friends/family
Quality of life—spiritual
Religion
Existential questioning
Impact on loved ones
Impact on loved ones:
Informal care
Acceptance
Work ability
Positive attitute
Psychological impact
Sexual relation
Social functioning
Future perspective
Being involved
Change in mentality
Relationship with partner
Societal impact
Societal costs:
Work ability
Volunteering work ability
(In)direct healthcare costs
Costs for employers
Ability to provide informal care
Payment benefits
Equality
Societal balancing
Cost‐effectiveness
Necessity
Implementation feasibility
Accesibility of care
Prevalence of disease
Freedom of choice
Innovation
Knowledge development
Quality of treatment
Quality of care:
Effectiveness
Long term health
Survival
Prognosis/progression
Patient centered
Ease of use
Side effects
Complications
Safety
Therapy compliance
Best possible care

**TABLE 3:** Quotes from the interviews

**Impact on daily life and future**
‘He was ill for a year and then he was still at the company and then it was about leasing a car. You have to do that for 4 or 5 years. Can we? And should we do that?’ (Partner of patient)
‘Well, of course you look at people as a whole and their participation in society so it's not only the cost but whether people can go back to their old work what they did before their illness? Can they still do that in the same way or are there many limitations to that? Do they feel they participate sufficiently in society? Doing sports or interacting with others in their social environment, are there limitations to that?’ (General practitioner)
**Patient costs**
‘People lose their jobs and therefore get into financial difficulties, but if you still need physiotherapy for medical complaints and you have to pay for those first 20 treatments yourself and you do not have that money. It also means that these financial problems actually hinder your recovery. And the same applies to psychological counseling, because that also costs money and that is often not insured or only very limited’. (Oncological nurse)
‘Statutory deductible premium recur every year. So, if you already had treatments in the hospital in 2019. And in 2020 you still have other complaints. Then you have to pay again every year’. (Health insurance company)
‘Loss of income because people can no longer work or have to look for other work. Is also a loss for the patient but also for society’ (Oncologist)
**Quality of life—physical**
‘Your physical condition can be permanently diminished, for example. Or you can move worse due to radiotherapy that you have had. So you will permanently have complaints about it. Or you will become infertile, maybe’. (Health insurance company)
‘Also physical limitations. If you entered the menopause early as a result of the treatment, you will also have all kinds of physical limitations if you are unlucky, which you also have to learn to deal with. Or if you have a very tight scar as a result of an operation that you feel continuously or that causes you to have a limitation of movement, then you may be able to do something about it with a treatment, but it may also be something you have to deal with for the rest of your life’. (Oncological nurse)
**Quality of life—psychological**
‘But also that you can sometimes psychologically change a bit because of medicines. You can become a bit unstable or have a different attitude. These are useful things to consider and at least tell patients’. (Oncological nurse)
‘You can remember endless sick beds in which people have gone to extremes and always have hope for just that marginal improvement and in the end the last months were an agony. You often notice that patients of course see a next therapy as a last straw to postpone death a bit and you do not want to take that straw away from them, while often you know in the back of your mind what a difficult road it will be with many side effects’. (General practitioner)
**Quality of life—social**
‘You see a lot of divorces, certainly with breast cancer. For example, because the partner does not grow along or, indeed, that things go wrong in the area of sex and intimacy. But also the people themselves change. The person changes and not everyone in the family can change accordingly’. (Primary care network)
‘Direct relationships of the patient can also suffer a lot of damage from a major event and treatment’. (Oncologist)
**Quality of life—spiritual**
‘Religion is also part of that, but also: how do you contribute to meaning in your life.” . “I often see this reflected in the quality of life questionnaires; that there are some essential questions in it, like spirituality. This is often not included in the psychological outcome measures such as emotions, fear and fear of recurrence. You can collect it within this but I think it deserves attention. That meaning and people's existential questions are simply very important’. (Oncologist)
**Impact on loved ones**
‘Of course it is also a lot for their husband or wife. If your partner has cancer and then important decisions have to be made. Of course you also have to go to the hospital and taxi often, so to speak. It takes you time too. How do you arrange that with your work? And perhaps with the care of children. How do you go about all that? It really has consequences for the whole family too’. (Health insurance company)
‘It is not at all that it affects us every day, it is of course always somewhere in the back of your mind and indeed it is always comes back with major decisions’. (Partner of patient)
**Societal impact**
‘In addition to survival, all other clinical outcomes that you see in the study are also taken into account, and quality of life is always included, at least there is always an attempt to take that into account. And in addition, a number of societal ones are also included in cost‐effectiveness analyses, such as productivity losses, the difference with quality losses’. … ‘And, for example, informal care and informal care costs are also included’. (National healthcare institute)
‘What we need to do is to be careful with health insurance deductible premiums. It's not just about sick people, it's also about young students who have nothing at all and have to pay deductibles. So you have to make sure that deductible premiums don't rise and get the most value with the money you have’. (Healthcare insurance company)
**Quality of treatment**
‘When we talk about new medicines, it's always about what to expect from them. Can you live longer, maybe you can heal? The first question is actually always, can I heal by it? Do I have a chance that I can heal by it? That has more impact than living one or two or three months longer. Does it offer a chance of survival, that has a lot of impact. If it offers no chance of survival but extension of life, then you would like to know with what qualities’. (Secondary care network)
‘Survival alone has of course long been an outcome of oncological care. It is a bit of a catch‐all term, but quality is really very important. Not because it is a luxury concept, but also because poor quality of life also entails high costs. People who are left with health damage from treatments who continue to seek help for that. Both with somatically oriented doctors and with other care providers, such as: physiotherapy, psychology’… ‘Survival is certainly not only important’. (Oncologist)

#### 3.1.1 Impact on daily life and future of patients

The interviewees mentioned several values that can be impacted by a cancer diagnosis or its treatment in daily or future life. Participants mentioned the ability to continue daily activities like sports, hobbies, doing one's own groceries or being independent. It was mentioned that patients can participate less in society and in activities such as working, volunteering, caring for children and being an informal caregiver. Participants stated that the importance of re‐integration or continuing work can differ per patient. Different participants mentioned the inability to make future plans and life choices. In addition, participants stated that mortgages are more difficult to get for (former) patients as they pay higher premiums or are rejected for life insurance, which they need to get a mortgage.

#### 3.1.2 Costs for patients and loved ones

The interviewees mentioned values regarding costs for patients and loved ones. A few participants stated that some additional healthcare or complementary care needs to be (partly) paid for by the patients themselves. Another cost aspect mentioned in the interviews was the deductible premium for the health insurance which is statutory in the Netherlands, even after treatments, because of a long‐term need for additional care. However, it was also stated that these problems differ per patient as it depends on a patient's personal financial situation.

Additional indirect costs for patients that were mentioned were loss of work and income. This problem also concerns partners as their ability to work might be affected as well (partly), because of caring for children, accompanying patients to the hospital or delivering informal care. Additionally, costs for re‐integration after patients have lost their job or for those who are self‐employed. Additional costs are spent on higher premiums for life insurance or disability insurance in case of self‐employment. Other examples are travel costs for patients and family members, house renovations, new wardrobes after weight loss or gain, wigs and sometimes prosthetics or specialized bras and swimwear.

#### 3.1.3 Quality of life

The interviewees mentioned values regarding the quality of life. Quality of life values is sub‐divided into physical, psychological, social and spiritual. It was mentioned that patients might be in need of support or companionship for these domains.

First, a cancer diagnosis can result in many physical consequences. It was mentioned that consequences differ depending on the treatment or tumour type. For instance, early menopause and infertility are common for patients with gynaecological cancers. For colon cancer, bowel dysfunction and stomas are common.

Second, participants stated that the psychological consequences can be quite severe. An often‐mentioned psychological effect is fear of death. Also, even after being cured, there is fear of recurrence or suffering from damage caused by cancer treatments. It was mentioned that some of the psychological impacts occurs after the passage of a certain time interval, that is when treatments are already completed. Psychological consequences might result in higher healthcare costs. Conflicting opinions existed about the value of hope. Hope can benefit a patient's quality of life but can also interfere with acceptance of the situation. In addition, different participants emphasized to focus more on positivity, fighting spirit and empowerment of patients instead of negative psychological consequences.

Third, participants state that social needs can be negatively influenced, for instance, by not attending social gatherings or not being energetic when a patient does attend a gathering. Additionally, the effect of these cancer treatments can affect patients' relationships.

Last, some spiritual aspects of quality of life were mentioned. Patients can find support in religion or existential questioning about the meaning of life.

#### 3.1.4 Impact on loved ones

The interviewees mentioned values regarding the impact on loved ones like psychological impact, workability, the positive or negative impact on relationships, social activities or the impact on future plans. Loved ones are often informal caregivers to patients. This entails being attentive to patients, driving patients to the hospitals and also providing care for patients at home. In addition, they can experience a change in mentality during the course of the disease. Loved ones might feel the need for support and companionship.

#### 3.1.5 Societal impact

Interviewees mentioned values regarding societal impact. The most frequently mentioned was the loss of productivity and lack of ability to return to work. These costs are borne by the patient (by not receiving wages), the employer and by society. In addition, costs are made on benefits and allowances paid to people losing their job or that are working less. Patients often have a certain capacity to work during the illness which is not always utilized. One participant mentioned the possibility of increased healthcare costs because of lower work productivity as it can affect a patient's psychological well‐being.

Other societal impacts mentioned were the loss of informal caregivers because the oncological patient cannot care for another family member or indirect healthcare costs on long‐term effects like heart diseases as a result of chemotherapy.

Participants often mention the importance of societal balancing of resource allocation as premium money paid on health insurance by society must be handled with caution and the most value for money should be created. Additional values that were mentioned to take into consideration were the prevalence of the disease, the development of new knowledge, innovation, freedom of choice for patients, access to care, equality, the necessity of a new drug and implementation feasibility.

#### 3.1.6 Quality of treatments

The interviewees mentioned values regarding the quality of treatments. It was often mentioned that patients should always get the best possible care and treatments should comply with the established medical sciences and practice. Besides survival or extending life, quality of life outcomes were deemed important. In addition, oncological treatments can cause serious long‐term health problems, like heart failure or other cancers. It was mentioned that side effects and ease of use of treatments should be proportional to the benefit. The ease of use includes, among others, the possibility and choice for care at home or the need for problems with transportation. Finally, it was mentioned that therapy compliance should be optimized.

### 3.2 Focus group with an expert panel

For patient‐level decision‐making, the extended exploration of values was deemed useful to ensure the inclusion of all values that count for patients in decision‐making that are important for a specific patient. However, the usefulness of many values also depends on the situation of the patient; and different aspects are important in different phases of the disease (i.e., palliative or curative), phase of treatment (i.e., when you start treatment or when you have a recurrence) and age of patients. It was mentioned that not all values are always relevant regarding choices of treatments (or not treating). The values were mentioned to be important to discuss with patients regarding long‐term effects and long‐term quality of life, for general information provision and empowerment of patients. In addition, the extended list of values was mentioned to be of importance in discussions on a patient's life after treatment or after cancer. It was mentioned that patient values are currently already being investigated to some extent during the treatment process, for instance, by means of questionnaires.

For reimbursement decisions, the use of the extended exploration of values was less clear. It was mentioned that the Netherlands has a mechanism of decision‐making using the QALY framework which is already incorporated many values. Other values are also considered, like the ease of use, although participants questioned whether national solidarity stretches as far as to reimburse treatments from national budgets due to them being specifically easier to use. Regarding the measurement methods of values, the expert panel mentioned that some values have established methods, while others do not.

Potential disadvantages of incorporating the extended exploration of values for reimbursement decisions were mentioned: (1) Risking an increase of the sustainability dilemma as more therapies might be reimbursed, (2) the uncertainty of how the value of, for example hope is proportional to benefits regarding survival and (3) the desire to use generic values that are applicable to all diseases instead of specific diseases.

Table 4 presents quotes from the expert panel.

**TABLE 4:** Quotes from the expert panel regarding decision‐making procedures

**Coverage and reimbursement decision‐making**
‘So I find it difficult, as you increase the list of values, that you allow more in the insurance package. Because then the sustainability dilemma will actually become even bigger’ (ZIN)
‘A value framework should in essence be generic, because only a value framework for oncology will not help us to keep healthcare sustainable’. (HTA expert)
‘If you have all that information. How do you include that in a decision‐making process? In other words. How are you going to weight it? And what belongs in which decision‐making process? It is not a question of the possibilities, but more a question of willingness’ … ‘We already have a working mechanism for what we think should be taken into account and how. The National Healthcare Institute has methods for that’. (HTA expert)
**Patient‐level decision‐making**
‘We do this in our hospital at the moment with every evaluation of the therapy, so when they have had a CT scan to see if things are going well or not. Then we discuss again with these patients what their values are and what they find important’… ‘The moment you are progressive and you have to make a choice for treating again, it is important to discuss with those patients whether a treatment will be useful’. (Oncologist)
‘What is that life with and after cancer? What is quality of life? And on which aspects? And who gives me control over that? And what do I need from both the healthcare professional and myself?’… ‘What do I need from society to ensure that I can still take out a mortgage? Or can participate? Or can continue to work? Or whatever. So there are a lot of questions that I think always belong in the treatment room, but are more difficult to weight at the population level’. (Patient association)

## 4 CONCLUSION AND DISCUSSION

Our findings reveal a broad range of values that matter to patients and involved stakeholders regarding new oncological treatments and decision‐making in oncological care. They can be categorized in (1) impact on daily life and future, (2) costs for patients and loved ones, (3) quality of life (physical, psychological, social and spiritual), (4) impact on loved ones, (5) societal impact and (6) quality of treatment.

Experts revealed the exploration of values was useful in patient‐level decision‐making. Not all values are always relevant to include in treatment choices, however, they can also be used for patient information and empowerment and support during and after treatment of cancer. The use of the extended exploration of values for reimbursement decisions was less clear.

### 4.1 Previous literature

Previous studies support the broad range of values that are relevant within oncology, going beyond existing value frameworks.

Our results suggest that decision‐making at the patient‐level should contain many values and patient preferences. Shared decision‐making (SDM) between physicians and patients can increase incorporation of patient preferences in treatment decisions, however, different studies reveal that SDM is not yet fully implemented and used to its full potential.^31^, ^32^, ^33^

Our results also suggest that not all values found in our study are included in reimbursement decisions. A review of US Value Frameworks by the ISPOR Value Special Task Force supports our findings as they state it is difficult for frameworks to represent values for all decision contexts.^34^ They mention that reimbursement decisions should be focused on efficiently allocating resources to maximize population health‐ and patient‐level decisions should include patients' values and preferences, within the larger constraints imposed by decisions on national levels.

Different studies found differences across countries regarding reimbursement decisions.^7^, ^35^, ^36^ A study by Angelis et al. examined differences in eight European countries regarding the assessment of the value of new medicines in the context of reimbursement decisions.^7^ Most countries implement a type of economic evaluation in addition to the assessment of clinical benefit. The preferred health gain measure usually is the QALY. Additional values beyond the QALY concept are captured to a different extent between countries, explaining some of the heterogeneity in reimbursement decisions. Ease of use, nature of the treatment, public health benefit, social productivity, place in therapeutic strategy and ethical considerations are criteria considered in some countries (either implicitly or explicitly) and not considered in others.

### 4.2 Comparison to existing value frameworks

Different frameworks exist for evaluating the value of health interventions. These frameworks can be generic or oncology specific and can inform reimbursement or patient‐level decisions. The added value of our findings to these existing frameworks is discussed below.

In the United States, the Institute for Clinical and Economic Review (ICER) has been evaluating the clinical and economic values of health interventions to aid reimbursement decisions.^37^ Their value framework incorporates long‐term value and short‐term affordability. Long‐term value includes incremental cost‐effectiveness and provides the possibility of incorporating additional other benefits or disadvantages and contextual considerations through deliberative processes. Short‐term affordability includes budget impact.

In Europe, the EUnetHTA published an HTA core model to aid reimbursement decisions for which different European countries collaborated.^38^ The ontology in this model covers the health problem and current use of technology, description and technical characteristics, safety, clinical effectiveness, costs and economic evaluation, ethical analysis, organizational aspect, patient and social aspects and legal aspects.

The ICER value framework and the HTA core model are extensive frameworks used for the systematic assessment of new treatments for reimbursement decisions, and our study can add to these frameworks by more explicitly using the values that are specific for oncological care in the deliberative processes.

Besides these models, two common oncological value frameworks are the American Society of Clinical Oncology (ASCO)^13^ and the European Society for Medical Oncology (ESMO)^9^ frameworks. These are used for facilitating shared decision‐making by patients and oncologists and could aid reimbursement decisions. The ASCO and ESMO incorporate clinical benefit, toxicity, QoL and improvement of (cancer‐related) symptoms beyond QoL. In addition, the ASCO adds treatment‐free interval, drug acquisition costs and patient co‐pay, and the ESMO adds daily well‐being, response rate and duration of response.

The value frameworks are used to aid physicians in explaining treatment benefits to patients and facilitate shared decision‐making. Our study adds to these cancer‐specific value frameworks by presenting explicit values regarding the impact on daily life and future, costs for patients and loved ones, QoL and quality of treatment. In addition, the impact on loved ones and societal impact are (mostly) lacking in these frameworks.

### 4.3 Strengths and limitations

The main strengths of this study are that we included a wide array of various stakeholders in the interviews and that multiple interviews were held per category, thus ensuring the capture of all the relevant perspectives. To our knowledge, this is the first study to explore a wider set of values in oncology.

We acknowledge some limitations. First, possibilities for incorporation of the values found in current decision‐making are only explored qualitatively. Case studies on oncological treatments will provide further insights into the true extent of how these values are included in decision‐making procedures. Second, our study did not include patients from all tumour types. Different patients with different tumour types could reveal additional values mainly for the physical and psychological quality of life. Third, as this study is performed in the Netherlands the results are mainly generalizable to different countries with a comparable healthcare setting (see Box 1 for a description). Finally, the aimal of the research was to explore value–elements for the assessment of new treatments. The themes represent those values, however, the subcategories within the main themes went beyond the scope of new treatment assessment based on the explorative and semi‐structured design of our interviews. Our approach ensures no mentioned values were missed and the focus group was used to assess the usefulness of this broad exploration of values. Despite these limitations, an overall idea of the broad range of values and their inclusion in decision‐making can be generated.

### 4.4 Conclusion and implications

In conclusion, clinical values are not the only ones that matter to oncological patients and involved stakeholders regarding the evaluation of new treatments and decision‐making procedures in oncological care. We found a much broader range of values. The recognition and appreciation of those values might add to patient‐level decision‐making, but the usefulness for reimbursement decisions is less clear. The values add to existing value frameworks used for both patient‐level and reimbursement decisions. In addition, the values might improve patient information, empowerment and support.

The results could be useful to guide clinicians and policymakers when it comes to decision‐making in oncology. We recommend exploring a more structural and explicit incorporation of values within oncology in patient‐level and reimbursement decision processes. At the patient level, the list of values can inform clinicians on which values to address in SDM, can be used for decision aids and can be used to provide extended patient information. For reimbursement decisions, it would be interesting to explore how the identified values can contribute. More research is needed on making explicit, for different oncological indications, if and how disease‐specific values can be systematically inventoried and incorporated to guarantee a more systematic approach to decision‐making and the deliberative processes.

## AUTHOR CONTRIBUTIONS

**Cilla E.J. Vrinzen:** Conceptualization (equal); data curation (lead); formal analysis (lead); investigation (lead); methodology (equal); project administration (equal); resources (equal); validation (equal); writing – original draft (lead). **Haiko J. Bloemendal:** Conceptualization (equal); methodology (equal); supervision (equal); validation (equal); writing – review and editing (equal). **Esra Stuart:** Formal analysis (equal); writing – review and editing (equal). **Amr Makady:** Conceptualization (equal); methodology (equal); writing – review and editing (equal). **Michel van Agthoven:** Conceptualization (equal); methodology (equal); writing – review and editing (equal). **Mariska Koster:** Conceptualization (equal); methodology (equal); writing – review and editing (equal). **Matthias AW Merkx:** Conceptualization (equal); methodology (equal); supervision (equal); writing – review and editing (equal). **Rosella P.M.G. Hermens:** Conceptualization (equal); investigation (equal); methodology (equal); supervision (equal); validation (equal); writing – review and editing (equal). **Patrick P.T. Jeurissen:** Conceptualization (equal); funding acquisition (equal); methodology (equal); supervision (equal); validation (equal); writing – review and editing (equal).

## FUNDING INFORMATION

This study is partly funded by Janssen‐Cilag BV, Breda, the Netherlands, pharmaceutical companies of Johnson & Johnson

## CONFLICT OF INTEREST

The study was partly funded by Janssen‐Cilag B.V., Breda, the Netherlands. CV, PJ, ES, MM, HB and RH have no conflict of interest to declare. AM, MK and MA are employees of Janssen‐Cilag B.V. and MA is a stock shareholder.

## ETHICAL APPROVAL

Ethical approval for non‐intrusive interview studies is not mandatory by Dutch law.

## Supporting information


Data S1

## Data Availability

The data that support the findings of this study are available on request from the corresponding author. The data are not publicly available due to privacy or ethical restrictions.
