# Supplementary material for: Cancer treatments touch a wide range of values that count for patients and other stakeholders: What are the implications for decision‐making?
Source: Cancer Med. 2022 Nov 14;12(5):6105–16. doi: 10.1002/cam4.5336 (PMC10028089; doi:10.1002/cam4.5336)
Supplement: Supplementary file 1 — Data S1 [file CAM4-12-6105-s001.docx]

**Appendix A: Interview topic guide**

Interview questions:

1. In the ideal situation in which there is access to all necessary data, which broad societal values are relevant to incorporate in the evaluation of new therapies?
2. Why these values?

Different categories found in literature are:

- Indirect or lang term costs (also for patients and loved ones)
- Societal costs
- Quality of life
- Impact in daily life
- Impact on loved ones
- Good quality of care

Additional interview questions regarding categories that are not already discussed in the first two questions:

1. Do you recognize [category] as a relevant for society?
2. Why?
3. Which values are relevant for you in this category?
4. Could you think of any more categories that are not mentioned yet?

(the topic guide contained some additional questions concerning a different research question)

**Appendix B: Literature study**

**Method**

A scoping review was carried out to identify societal values with oncology and their overarching theme’s. The final search strategy included keywords (Medical Subject Headings (MeSH-terms)), subject headings and free text terms reflecting the following topic areas: cancer/oncology, biomedical technology assessment and a broader/extended scope regarding value or outcome measures described. The search string contains different terms within the categories: "Cancer" and "Values" and "HTA, cost-effectiveness or societal". The search string is presented in Box S1. The search was performed in PubMed.

Studies were excluded if they were published before the year 1990. After the initial search, duplicates and articles that were published in languages other than English or Dutch were discarded. Studies were included in full text screening if title abstract screening showed potential for describing values beyond existing value frameworks and basic cost-effectiveness analyses. The studies of *Chandra et al* and *Campolina* present these frameworks and their included values.^14, 40^ Articles for which no full text was openly available were excluded for inaccessibility. During full text screening, studies were excluded if they: (1) were no empirical or (narrative) review study; (2) did not focus on a (partially) cancer-specific population; (3) mentioned no specific value elements; (4) only described value elements already mentioned in existing oncological value frameworks.^14, 40^

Screening of titles and abstracts was done individually by one independent researcher. However, when in doubt, consolations and discussions were held with a second researcher. Two researchers simultaneously worked on screening the full text articles. In cases of doubt about in-/ or exclusion of studies, studies were labeled, assessed again by both researchers independently and discussed until consensus was reached. Grey literature search beyond traditional academic publishing was performed by searching websites of relevant Dutch organizations.

Study characteristics and outcomes were extracted from all included studies. One researcher identified outcomes of broad/societal value with Atlas.ti (version 8). Identified values were categorized in overarching themes.

Box S1. Pubmed search string

| ("Neoplasms/therapy"[Mesh] OR Oncolog*[tiab] OR cancer [tiab])  AND  ("Technology Assessment, Biomedical"[Mesh:NoExp] OR "economics" [Subheading] OR HTA[tiab] OR Health technology assessment*[tiab] OR cost-effect*[tiab] OR cost-analys*[tiab] OR cost-benefit analys*[tiab] OR societal[tiab] OR social[tiab])  AND  ( (Value*[ti])  OR  ( (expanding[ti] OR expanded[ti] OR extending [ti] OR extended [ti] OR broader[ti] OR broad[ti] OR future[ti]) AND (HTA[ti] OR Health technology assessment*[ti] OR cost*[ti] OR societal[ti] OR social[ti]) ) )  *Filters: from 1990* |
| --- |

**Results**

A total of 900 studies were identified through PubMed search. Two records were excluded manually as duplicates, as these were re-publication done by the same researchers, describing identical results following identical methodology. After TIAB and full text screening, 19 articles from the primary search remained for final inclusion. Five reports were identified as relevant grey literature, adding up to the total of 24 articles included in this scoping review (Figure S1). An extensive summary of the included studies' characteristics is provided in Table S1.

Table S2 presents all values derived from the articles included in this review. Categorization of results led to eleven overarching themes: (1) impact on daily life and future, (2) health related outcomes, (3) patient and caregiver costs, (4) family burden, (5) quality of life in general, (6) quality of life – physical, (7) quality of life – psychological, (8) quality of life – social, (9) quality of life – spiritual, (10) societal, and (11) quality of care regarding care/treatment.


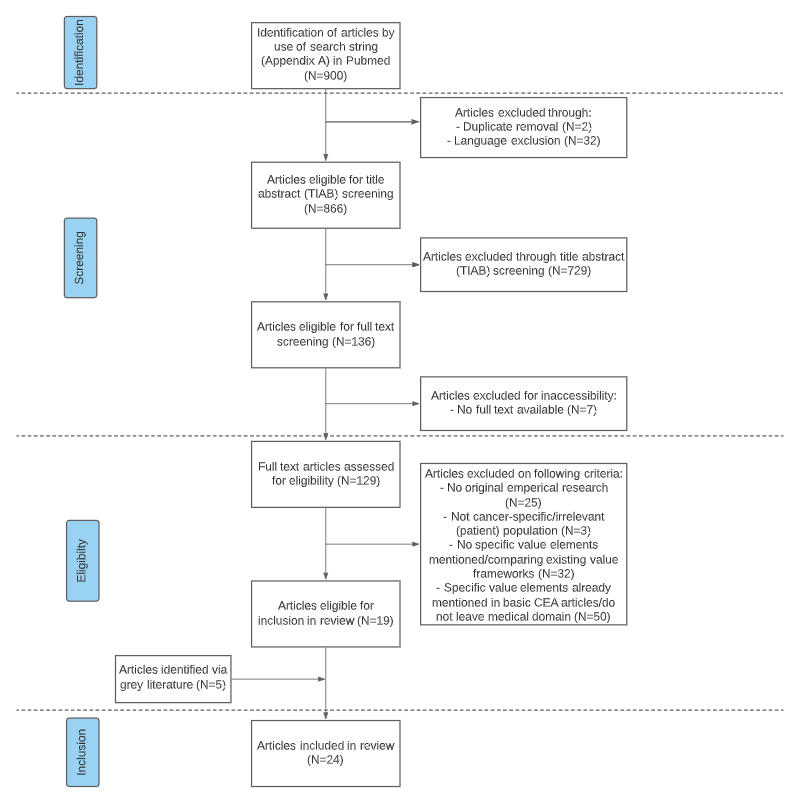


Figure S1. Flowdiagram of study inclusions

Table S1. Characteristics of the included studies

| **Authors** | **Year** | **Country** | **Study population** | **Goal** | **Study Type** |
| --- | --- | --- | --- | --- | --- |
| Davies^41^ | 1996 | UK | Patients with malignant cerebral glioma and their caregivers | Exploring perspectives of patients and relatives on the value of radiotherapy | Interview study |
| dosReis. ^24^ | 2020 | USA | Patient representative members of PAVE Stakeholder Advisory Committee (SAC); patient stakeholder community representatives | Stakeholder-Engaged Derivation of Patient-Informed Value Elements | Mixed-methods |
| Ersek^42^ | 2018 | USA | Metastatic lung cancer patients | Exploring clinical pathways and patient perspective in the pursuit of value-based oncology care | Review |
| Gidwani-Marszowski^43^ | 2018 | USA | Oncologists | Exploring oncologists' views on using value to guide cancer treatment decisions | Interview study |
| Hughes^44^ | 2019 | UK | Studies into hospice patients and/or their caregivers | Exploring value of patients and family-caregivers regarding hospice care | Systematic review |
| Jarrett^45^ | 2013 | UK | Cancer survivors | Reviewing psychological and social problems faced by cancer survivors | Rapid review |
| Johnson^46^ | 2017 | Australia | Metastatic cancer patients and family | Exploring values of patients and their families regarding at the end of life | Interview study |
| Kaufman^47^ | 2019 | USA | Immuno-oncology treatment receiving cancer patients | Reviewing the promise of immuno-oncology and the implications for defining the value | Narrative review |
| Kim^48^ | 2012 | Worldwide | Caregivers of patients with various cancer types | Reviewing the value and needs of the caregiver in oncology | Review |
| Konski^49^ | 2017 | USA | Radiation oncology patients | Reviewing value in radiation oncology and approaches to weighing benefits vs costs | Narrative review |
| Lakdawalla^25^ | 2012 | USA | Cancer patients | Exploring how cancer patients value hope and the implications for cost-effectiveness assessments of high-cost cancer therapies | Survey and interview study |
| Lorgelly^29^ | 2020 | UK | Cancer patients | Exploring what outcomes patient with cancer value in outcome-based payment schemes | Mixed-methods |
| Mitchell^50^ | 2020 | USA | Cancer patients | Reviewing needs, values, and preferences among adult patients during their cancer treatment and identifying important components of patient-centered cancer care | Systematic review of qualitative studies |
| Nardi^51^ | 2016 | USA | NCCN Work Group | Examining the challenges of access, high costs, and defining and demonstrating value at the academic cancer centers. | (Work group) report |
| Sacristán^52^ | 2016 | Spain | Oncologists, healthcare policy makers, patients, individuals from the general population | Exploring the main factors explaining the relative weight of the attributes that determine the value of oncologic treatments from different perspectives. | Interview study |
| Shafrin^53^ | 2018 | Canada | Patients with squamous non-small cell lung cancer (NSCLC) | Exploring the impact of expanding cost-effectiveness analysis for second line nivolumab for patients with squamous non-small cell lung cancer | Cost-effectiveness analysis |
| Teckie^54^ | 2014 | USA | Radiation oncology patients | Reviewing the meaning of value, the current economic landscape and impediments to achieving value | Narrative review |
| Tseng^55^ | 2016 | Canada | - | Reviewing the differences between value-based care and patient-centered care | Narrative review |
| Verdonck-de Leeuw^56^ | 2012 | The Netherlands | Head and neck cancer patients (and caregivers) | Exploring the value of quality-of-life questionnaires in head and neck cancer | Review |
|  |  |  |  |  |  |
| *Grey literature* | |  |  |  |  |
| IKNL ^57^ | 2019 | The Netherlands | (Ex-)cancer patients | Exploration of the broad consequences of cancer | Secondary analysis of existing data |
| NIVEL^58^ | 2005 | The Netherlands | (Ex-)cancer patients | Exploring the healthcare and societal situation of people with cancer | Mixed method |
| NIVEL^59^ | 2010 | The Netherlands | (Ex-)cancer patients | Exploring the need of support of (ex)cancer patient and the role of the general practitioner | Questionnaire study |
| NIVEL^60^ | 2013 | The Netherlands | (Ex-)cancer patients | Exploration of experienced problems and support need van cancer patients | Questionnaire study |
| RIVM^61^ | 2016 | The Netherlands | (Ex-)cancer patients | Exploring and informing about cancer, cancer care, cancer patients and society | Narrative review and exploration of different secondary data sources |

Table S2. Values from literature

| Themes | Studies |
| --- | --- |
| General health related outcome | DosReis, Ersek, Gidwani-Marszowski, Kaufman, Konski, Lorgelly, Nardi, NIVEL (2013), Teckie, Tseng, |
| Daily life and future | Davies, DosReis, Ersek, Gidwani-Marszowski, IKNL, Johnson, Kaufman, Mitchell, NIVEL (2005), NIVEL (2010), NIVEL (2013), Lorgelly, RIVM, Sacristan, Shafrin, Tseng |
| Patient and caregiver costs | DosReis, Ersek, Gidwani-Marszowski, Johnson, Kaufman, Konski, Nardi, Shafrin, Teckie, Tseng, IKNL, NIVEL(2005), NIVEL(2010), NIVEL(2013) |
| Family burden | Davies, DosReis, Gidwani-Marszowski, Hughes, Jarret, Johnson, Kim, Lorgelly, Mitchell, Tseng, Shafrin, RIVM, Verdonck - de Leeuw |
| Quality of life in general | Ersek , Gidwani-Marszowski, Kaufman, Konski, Nardi, Sacristan, Jarret, Shafrin, NIVEL (2005) |
| Quality of life - Physical | Davies, DosReis, Ersek, IKNL, Kaufman, Konski, Lorgelly, Nardi, NIVEL (2005), NIVEL (2010), NIVEL (2013), Sacristan, Shafrin, Teckie, Tseng, Verdonck - de Leeuw |
| Quality of life - Psychological | Davies, DosReis, Ersek, Gidwani-Marszowski, Hughes, IKNL, Jarret, Johnson, Kaufman, Lakdawalla, Lorgelly, Mitchell, NIVEL (2005), NIVEL (2010), NIVEL (2013), RIVM, Shafrin, Teckie, Tseng, Verdonck - de Leeuw |
| Quality of life - Social | Davies, DosReis, Hughes, IKNL, Jarret, Johnson, Lorgelly, Jarret, Teckie, Mitchell, NIVEL(2005), NIVEL(2010), NIVEL(2013), RIVM |
| Quality of life - Spiritual | DosReis, Ersek, Tseng, NIVEL (2013) |
| Societal | DosReis, Ersek, Gidwani-Marszowski, IKNL, Kaufman, Kim, Konski, Lorgelly, Nardi, NIVEL(2005), NIVEL (2010) RIVM, Sacristan, Shafrin, Teckie, Tseng, |
| Quality of care | Davies, DosReis, Ersek, Gidwani-Marszowski, IKNL, Johnson, Hughes, Kaufman, Kim, Konski, Lorgelly, Mitchell, Nardi, NIVEL (2005), NIVEL (2010), NIVEL (2013), RIVM, Sacristan, Teckie, Tseng, Verdonck - de Leeuw, Teckie |

## **References**

1. World Health Organization (WHO). Cancer Tomorrow. A tool that predicts the future cancer incidence and mortality burden worldwide from the current estimates in 2020 up until 2040. 2020 [Available from: <https://gco.iarc.fr/tomorrow/>. .

2. Hofmarcher T, Lindgren P, Wilking N, Jönsson B. The cost of cancer in Europe 2018. Eur J Cancer. 2020;129:41-9.

3. Cliff B. The evolution of patient-centered care. J Healthc Manag. 2012;57(2):86-8.

4. Ekman I, Ebrahimi Z, Olaya Contreras P. Person-centred care: looking back, looking forward. Eur J Cardiovasc Nurs. 2021;20(2):93-5.

5. Ekman I, Swedberg K, Taft C, Lindseth A, Norberg A, Brink E, et al. Person-centered care--ready for prime time. Eur J Cardiovasc Nurs. 2011;10(4):248-51.

6. Rawson JV, Moretz J. Patient- and Family-Centered Care: A Primer. J Am Coll Radiol. 2016;13(12 Pt B):1544-9.

7. Angelis A, Lange A, Kanavos P. Using health technology assessment to assess the value of new medicines: results of a systematic review and expert consultation across eight European countries. Eur J Health Econ. 2018;19(1):123-52.

8. European Network for Health Technology Assessment Joint Action 2 (EUnetHTA JA 2). HTA Core Model Version 3.0. 2016.

9. Cherny NI, Sullivan R, Dafni U, Kerst JM, Sobrero A, Zielinski C, et al. A standardised, generic, validated approach to stratify the magnitude of clinical benefit that can be anticipated from anti-cancer therapies: the European Society for Medical Oncology Magnitude of Clinical Benefit Scale (ESMO-MCBS). Ann Oncol. 2015;26(8):1547-73.

10. Drug Pricing Lab MSKCC. Drug Abacus [Available from: <https://drugpricinglab.org/>.

11. National Comprehensive Cancer Network. NCCN Clinical Practice Guidelines in Oncology (NCCN Guidelines) with NCCN Evidence Blocks 2021 [Available from: <https://www.nccn.org/guidelines/guidelines-with-evidence-blocks>.

12. Schnipper LE, Davidson NE, Wollins DS, Tyne C, Blayney DW, Blum D, et al. American Society of Clinical Oncology Statement: A Conceptual Framework to Assess the Value of Cancer Treatment Options. J Clin Oncol. 2015;33(23):2563-77.

13. Schnipper LE, Davidson NE, Wollins DS, Blayney DW, Dicker AP, Ganz PA, et al. Updating the American Society of Clinical Oncology Value Framework: Revisions and Reflections in Response to Comments Received. Journal of Clinical Oncology. 2016;34(24):2925-34.

14. Campolina AG. Value-based medicine in oncology: the importance of perspective in the emerging value frameworks. Clinics (Sao Paulo). 2018;73(suppl 1):e470s.

15. Chandra A, Shafrin J, Dhawan R. Utility of Cancer Value Frameworks for Patients, Payers, and Physicians. Jama. 2016;315(19):2069-70.

16. Cohen JT, Anderson JE, Neumann PJ. Three Sets of Case Studies Suggest Logic and Consistency Challenges with Value Frameworks. Value Health. 2017;20(2):193-9.

17. Slomiany M, Madhavan P, Kuehn M, Richardson S. Value Frameworks in Oncology: Comparative Analysis and Implications to the Pharmaceutical Industry. Am Health Drug Benefits. 2017;10(5):253-60.

18. Kaufman HL, Atkins MB, Dicker AP, Jim HS, Garrison LP, Herbst RS, et al. The Value of Cancer Immunotherapy Summit at the 2016 Society for Immunotherapy of Cancer 31st Anniversary Annual Meeting. Journal for ImmunoTherapy of Cancer. 2017;5(1):38.

19. Krzyszczyk P, Acevedo A, Davidoff EJ, Timmins LM, Marrero-Berrios I, Patel M, et al. The growing role of precision and personalized medicine for cancer treatment. Technology (Singap World Sci). 2018;6(3-4):79-100.

20. Garrison LP, Jr., Kamal-Bahl S, Towse A. Toward a Broader Concept of Value: Identifying and Defining Elements for an Expanded Cost-Effectiveness Analysis. Value Health. 2017;20(2):213-6.

21. Lakdawalla DN, Doshi JA, Garrison LP, Jr., Phelps CE, Basu A, Danzon PM. Defining Elements of Value in Health Care-A Health Economics Approach: An ISPOR Special Task Force Report [3]. Value Health. 2018;21(2):131-9.

22. Longworth L, Yang Y, Young T, Mulhern B, Hernández Alava M, Mukuria C, et al. Use of generic and condition-specific measures of health-related quality of life in NICE decision-making: a systematic review, statistical modelling and survey. Health Technol Assess. 2014;18(9):1-224.

23. Caro JJ, Brazier JE, Karnon J, Kolominsky-Rabas P, McGuire AJ, Nord E, et al. Determining Value in Health Technology Assessment: Stay the Course or Tack Away? Pharmacoeconomics. 2019;37(3):293-9.

24. dosReis S, Butler B, Caicedo J, Kennedy A, Hong YD, Zhang C, et al. Stakeholder-Engaged Derivation of Patient-Informed Value Elements. Patient. 2020;13(5):611-21.

25. Lakdawalla DN, Romley JA, Sanchez Y, Maclean JR, Penrod JR, Philipson T. How cancer patients value hope and the implications for cost-effectiveness assessments of high-cost cancer therapies. Health Aff (Millwood). 2012;31(4):676-82.

26. Kim H, Goodall S, Liew D. Health Technology Assessment Challenges in Oncology: 20 Years of Value in Health. Value Health. 2019;22(5):593-600.

27. Tong A, Sainsbury P, Craig J. Consolidated criteria for reporting qualitative research (COREQ): a 32-item checklist for interviews and focus groups. International Journal for Quality in Health Care. 2007;19(6):349-57.

28. Kroneman M, Boerma W, van den Berg M, Groenewegen P, de Jong J, van Ginneken E. Netherlands: Health System Review. Health Syst Transit. 2016;18(2):1-240.

29. Lorgelly P, Pollard J, Cubi-Molla P, Cole A, Sim D, Sussex J. Outcome-Based Payment Schemes: What Outcomes Do Patients with Cancer Value? Patient. 2020;13(5):599-610.

30. Mitchell KR, Brassil KJ, Rodriguez SA, Tsai E, Fujimoto K, Krause KJ, et al. Operationalizing patient-centered cancer care: A systematic review and synthesis of the qualitative literature on cancer patients' needs, values, and preferences. Psychooncology. 2020;29(11):1723-33.

31. de Ligt KM, van Egdom LSE, Koppert LB, Siesling S, van Til JA. Opportunities for personalised follow-up care among patients with breast cancer: A scoping review to identify preference-sensitive decisions. Eur J Cancer Care (Engl). 2019;28(3):e13092.

32. Meijers MC, Noordman J, Spreeuwenberg P, Olde Hartman TC, van Dulmen S. Shared decision-making in general practice: an observational study comparing 2007 with 2015. Fam Pract. 2019;36(3):357-64.

33. Spinnewijn L, Aarts J, Verschuur S, Braat D, Gerrits T, Scheele F. Knowing what the patient wants: a hospital ethnography studying physician culture in shared decision making in the Netherlands. BMJ Open. 2020;10(3):e032921.

34. Willke RJ, Neumann PJ, Garrison LP, Jr., Ramsey SD. Review of Recent US Value Frameworks-A Health Economics Approach: An ISPOR Special Task Force Report [6]. Value Health. 2018;21(2):155-60.

35. Angelis A, Lange A, Kanavos P. Using health technology assessment to assess the value of new medicines: results of a systematic review and expert consultation across eight European countries. The European Journal of Health Economics. 2018;19.

36. Clement FM, Harris A, Li JJ, Yong K, Lee KM, Manns BJ. Using effectiveness and cost-effectiveness to make drug coverage decisions: a comparison of Britain, Australia, and Canada. Jama. 2009;302(13):1437-43.

37. Thokala P, Carlson JJ, Drummond M. HTA'd in the USA: A Comparison of ICER in the United States with NICE in England and Wales. J Manag Care Spec Pharm. 2020;26(9):1162-70.

38. Institute for Clinical and Economic Review. 2020-2023 Value Assessment Framework. 2020.

39. EUnetHTA. Joint Action on HTA 2012-2015, HTA Core Model version 3.0 2016.

40. Chandra A, Shafrin J, Dhawan R. Utility of Cancer Value Frameworks for Patients, Payers, and Physicians. JAMA. 2016;315(19):2069-70.

41. Davies E, Clarke C, Hopkins A. Malignant cerebral glioma--II: Perspectives of patients and relatives on the value of radiotherapy. Bmj. 1996;313(7071):1512-6.

42. Ersek JL, Nadler E, Freeman-Daily J, Mazharuddin S, Kim ES. Clinical Pathways and the Patient Perspective in the Pursuit of Value-Based Oncology Care. Am Soc Clin Oncol Educ Book. 2017;37:597-606.

43. Gidwani-Marszowski R, Nevedal AL, Blayney DW, Patel M, Kelly PA, Timko C, et al. Oncologists' Views on Using Value to Guide Cancer Treatment Decisions. Value Health. 2018;21(8):931-7.

44. Hughes NM, Noyes J, Eckley L, Pritchard T. What do patients and family-caregivers value from hospice care? A systematic mixed studies review. BMC Palliat Care. 2019;18(1):18.

45. Jarrett N, Scott I, Addington-Hall J, Amir Z, Brearley S, Hodges L, et al. Informing future research priorities into the psychological and social problems faced by cancer survivors: a rapid review and synthesis of the literature. Eur J Oncol Nurs. 2013;17(5):510-20.

46. Johnson SB, Butow PN, Kerridge I, Tattersall MH. What do patients with cancer and their families value most at the end of life? A critical analysis of advance care planning. Int J Palliat Nurs. 2017;23(12):596-604.

47. Kaufman HL, Atkins MB, Subedi P, Wu J, Chambers J, Joseph Mattingly T, 2nd, et al. The promise of Immuno-oncology: implications for defining the value of cancer treatment. J Immunother Cancer. 2019;7(1):129.

48. Kim Y, Carver CS. Recognizing the value and needs of the caregiver in oncology. Curr Opin Support Palliat Care. 2012;6(2):280-8.

49. Konski AA. Defining Value in Radiation Oncology: Approaches to Weighing Benefits vs Costs. Oncology (Williston Park). 2017;31(4):248-54.

50. Mitchell KR, Brassil KJ, Rodriguez SA, Tsai E, Fujimoto K, Krause KJ, et al. Operationalizing patient-centered cancer care: A systematic review and synthesis of the qualitative literature on cancer patients' needs, values, and preferences. Psychooncology. 2020.

51. Nardi EA, Wolfson JA, Rosen ST, Diasio RB, Gerson SL, Parker BA, et al. Value, Access, and Cost of Cancer Care Delivery at Academic Cancer Centers. J Natl Compr Canc Netw. 2016;14(7):837-47.

52. Sacristán JA, Lizan L, Comellas M, Garrido P, Avendaño C, Cruz-Hernández JJ, et al. Perceptions of Oncologists, Healthcare Policy Makers, Patients and the General Population on the Value of Pharmaceutical Treatments in Oncology. Adv Ther. 2016;33(11):2059-68.

53. Shafrin J, Skornicki M, Brauer M, Villeneuve J, Lees M, Hertel N, et al. An exploratory case study of the impact of expanding cost-effectiveness analysis for second-line nivolumab for patients with squamous non-small cell lung cancer in Canada: Does it make a difference? Health Policy. 2018;122(6):607-13.

54. Teckie S, McCloskey SA, Steinberg ML. Value: a framework for radiation oncology. J Clin Oncol. 2014;32(26):2864-70.

55. Tseng EK, Hicks LK. Value Based Care and Patient-Centered Care: Divergent or Complementary? Curr Hematol Malig Rep. 2016;11(4):303-10.

56. Verdonck-de Leeuw IM, van Nieuwenhuizen A, Leemans CR. The value of quality-of-life questionnaires in head and neck cancer. Curr Opin Otolaryngol Head Neck Surg. 2012;20(2):142-7.

57. Integraal Kankercentrum Nederland (IKNL). Kankerzorg in beeld: over leven met en na kanker. IKNL, NFK; 2019.

58. NIVEL. Zorg- en maatschappelijke situatie van mensen met kanker in Nederland. 2005.

59. NIVEL. Ondersteuningsbehoeften van (ex-)kankerpatiënten en de rol van de huisarts. 2010.

60. NIVEL. Deelrapportage I: Ervaren problemen en ondersteuningsbehoeften van

mensen met kanker. NIVEL, IKNL; 2013.

61. Rijksinstituut voor Volksgezondheid en Milieu (RIVM). Een samenhangend beeld van kanker: ziekte, zorg, mens en maatschappij. 2016.

**Appendix C: Topic guide expert panel**

Questions for the focus group with an expert panel:

1. What is the desire for this broad value framework for evaluation and decision-making regarding oncological therapies? Within which context is this desirable? Why?
2. With which methodologies can these values be incorporated in decision-making processes? And how will this look like?
3. What is the current use of these values in evaluations and decision-making processes?
